# Somatic symptom load in men and women from middle to high age in the Gutenberg Health Study - association with psychosocial and somatic factors

**DOI:** 10.1038/s41598-019-40709-0

**Published:** 2019-03-14

**Authors:** Manfred E. Beutel, Jörg Wiltink, Jasmin Ghaemi Kerahrodi, Ana N. Tibubos, Elmar Brähler, Andreas Schulz, Philipp Wild, Thomas Münzel, Karl Lackner, Jochem König, Norbert Pfeiffer, Matthias Michal, Michaela Henning

**Affiliations:** 1 Department of Psychosomatic Medicine and Psychotherapy, University Medical Center, Johannes Gutenberg-University, Mainz, Germany; 2 Preventive Cardiology and Preventive Medicine - Center for Cardiology, University Medical Center Mainz, Mainz, Germany; 3 Center for Cardiology - Cardiology I, University Medical Center Mainz, Mainz, Germany; 4 Institute of Medical Biostatistics, Epidemiology and Informatics, University Medical Center of the Johannes Gutenberg-University Mainz, Mainz, Germany; 5 Institute of Clinical Chemistry and Laboratory Medicine, University Medical Center of the Johannes Gutenberg-University Mainz, Mainz, Germany; 6 Department of Ophthalmology, University Medical Center Mainz of the Johannes Gutenberg-University Mainz, Mainz, Germany; 7 Center for Thrombosis and Hemostasis, University Medical Center of the Johannes Gutenberg-University Mainz, Mainz, Germany; 8 DZHK (German Center for Cardiovascular Research), partner site Rhine-Main, Mainz, Germany; 9 Center for Translational Vascular Biology (CTVB), University Medical Center of the Johannes Gutenberg-University Mainz, Mainz, Germany; 10 Department of Psychosomatics and Psychotherapy, University Hospital Cologne, University of Cologne, Cologne, Germany

## Abstract

The purpose of the study was (1) to determine the prevalence of somatic symptoms in men and women in the general population and (2) to identify the contributions of psychosocial factors and somatic disease on symptom reporting. A total of 7,925 participants aged 40 to 80 years underwent medical and psychological assessments, based on the PHQ-15 (Patient Health Questionnaire). We excluded 3 items in order to avoid confounding findings: 2 items overlapping with the depression measure (PHQ-9) and the menstruation complaints item which biases sex comparisons. Pain complaints (arms, legs, joints, back pain) affected the majority of men and women, and somatic symptom reporting increased with age. When confounding has been reduced, psychosocial factors (lack of social support, adverse life events, loneliness, depression, generalized anxiety, panic, social phobia) have remained the strongest predictors of somatic symptoms. As shown by the interaction between sex and depression, depression plays a smaller role for somatic symptom reporting in women vs. men. Findings highlight the complex psychosocial and somatic contributions to somatic symptom reporting.

## Introduction

Somatic symptom burden in the German population had already been subject of previous studies^1,2^ but our aim is to assess both, the somatic determinants of symptom reporting without confounding of the symptoms of depression and the psychosocial determinants. In a biopsychosocial model we included somatic diseases, psychological factors and potential social determinants of somatic symptom reporting. This will be indispensable as a foundation for accurate assessment of the new diagnostic entity of “somatic symptom disorder” which has been introduced in the last version of the DSM V.

A high somatic symptom burden has been related to reduced subjective health and quality of life, and to increased psychological distress and use of health care services^3–5^. There is a high prevalence of somatic symptoms not only in primary care patients^6^, but also in the general population^5^. A persistent burden of bodily symptoms without sufficient medical explanation or pathology has been termed medically unexplained illness^7^, functional somatic syndrome^8^, somatization disorder, or – in the latest version of the DSM V - somatic symptom disorder, when combined with high levels of health-related preoccupation and anxiety^9^. In a representative survey of the German population, Kocalevent *et al*.^2^ reported a prevalence of somatization disorder of 9.3% in the German population (age 14–92 years) based on the PHQ-15 (> = 10) (Patient Health Questionnaire)^10^. Based on the same criteria, higher prevalences were reported by Hinz *et al*.^1^ from the LIFE cohort from Leipzig (age range 18 to 80 years): total somatization disorder 11.7%; Men 7.4%, women 15.6%. The majority of participants suffered from the following somatic complaints: sleep problems (women: 76, men 59.6%); back pain (women: 67.4%; men 59.3%); tiredness (women 67.53%; men 57.1%) and pain in arms, legs and joints (women 61.7%; men 54.6%).

Somatic symptom burden has been associated with *sociodemographic factors* of higher age, lower education, social and economic status, unemployment^1–3,11^ and disruption of relationships by separation, divorce or widowhood^3,12^.

In non-patient and in medical populations, women commonly reported more frequently chronic and intense symptoms than men, and women’s prevalence of somatization was considerably higher^13,14^. However, reasons for the sex differential appear to be complex. These have been attributed to 1.) women’s higher prevalence of common mental disorders with a strong somatic component, esp. depression and anxiety, 2.)  their higher rates of current and past abuse and trauma, and 3.) women’s tendency to judge and describe physical sensations as more bothersome and report them to a physician. This reporting bias has been attributed both to innate differences in nociception and to gender-specific socialization with a higher willingness to acknowledge and communicate distress compared to men. Comparing men and women on the basis of the PHQ-15, previous studies have not considered the bias that women can be expected to have higher scores based on the fact that they fill in one more item (menstrual complaints) than men.

Less is known about the role of *behavioral factors*: high rates of unhealthy behavior have been shown in psychotherapy inpatients and outpatients, including somatization disorder^15^.

Among the psychosocial factors, there has been a linear relationship between the number of physical symptoms reported and the degree of psychological distress. A strong overlap between somatization, depression and anxiety disorders has been found both in primary care patients and in the general population^6^. However, the association has been conflated by the overlap of two items between PHQ-9 and PHQ-15. The PHQ-9 is a self-administered diagnostic instrument for depression which scores each of the diagnosis criteria from the Diagnostic and Statistical Manual of Mental Disorders, Fourth Edition (DSM-IV) from 0 (“not at all”) to 3 (“nearly every day”). Kroenke *et al*.^16^ reported a high sensitivity and specificity for major depression but also show that the PHQ-9 is a reliable and valid measure of depression severity. The PHQ-15 is a screening instrument for the most prevalent DSM-IV somatization disorder somatic symptoms. Subjects rate the severity of 13 symptoms as 0 (“not bothered at all”) to 1 (“bothered a little”) or 2 “bothered a lot”). “Feeling tired or having little energy” and “trouble sleeping” are the two items that are contained in both PHQ-9 and PHQ-15 and are rated as described in the PHQ-9 from 1 to 3.

Loneliness has been associated with poor physical health and an increased mortality. Less, however, is known about the association between loneliness and somatic symptom reporting. A recent study by Le Roy *et al*.^17^ found that loneliness predicted more symptoms of a common cold after an experimental viral infection.

Somatic symptoms were highly prevalent in the majority of cancer patients^18^; somatic symptom load was strongly associated with disability in cancer^18^, in COPD^19^, in cardiac^20^ and in primary care patients^6^. In primary care, in the majority of cases there was an overlap of somatization with depression and anxiety^21^. The relationship between symptoms and objective physiological parameters has been strong in acute disease (e.g. pain), but moderate and highly variable in chronic and multi-symptomatic diseases^22^.

In their systematic review of 40 somatic symptom questionnaires, Zijlema *et al*.^23^ recommended the PHQ-15 as a psychometrically sound and internationally available measure. In order to avoid confounding with depression, we used the 12 item version of the PHQ, leaving out the two items overlapping with the PHQ-9: feeling tired or having little energy and trouble sleeping. To ensure comparability between men and women, we also left out the single item on menstrual complaints. Due to the age structure of the large population sample of the general population from middle (40 years) to old age (80 years), we expected a considerable somatic symptom load due to the age-associated increase of chronic cardiovascular, metabolic, pulmonary disease and cancer. Our aims were:

1. To assess the prevalence of somatic symptoms in the general population
2. To identify psychosocial and somatic factors associated with a high somatic symptom load.

We expected a higher somatic symptom load in men and in women with (a) social risk factors, (b) cardiovascular risk, (c) mental distress (depression, anxiety, life events, loneliness), (d) somatic disease (CVD, cancer, etc.). We expected mental distress to be the strongest risk factor.

## Material and Methods

### Procedure and study sample

The Gutenberg Health Study (GHS) is a population-based, prospective, observational single-center cohort study in the Rhine-Main-Region, Germany. Its primary aim is to analyze and improve cardiovascular risk factors and their stratification. The study protocol and documents were approved by the local ethics committee of the Medical Chamber of Rhineland-Palatinate and the local data safety commissioner and all research was performed in accordance with relevant guidelines and regulations. Participants were included after informed consent. Insufficient knowledge of German language, psychological or physical impairment with regard to participation led to exclusion. All study investigations have been conducted in line with the Declaration of Helsinki and principles outlined in recommendations for Good Clinical Practice and Good Epidemiological Practice. The sample was drawn randomly from the local registry in the city of Mainz and the district of Mainz-Bingen. The sample was stratified 1:1 for gender and residence and in equal strata for decades of age. Inclusion criteria were age 35 to 74 years and written informed consent. The response rate (defined as the recruitment efficacy proportion, i.e. the number of persons with participation in or appointment for the baseline examination divided by the totaled number of persons with participation in or appointment for the baseline examination plus those with refusal and those who were not contactable) was 60.3%. The design and the rationale of the Gutenberg Health Study (GHS) have been described in detail elsewhere^24^.

At baseline, a total of 15,010 participants were examined between 2007 and 2012. This study was based on the follow-up data of the first 10.000 participants who were reexamined after five years (participation rate of 81%). N = 7,974 of the baseline sample filled out the PHQ-15 at follow-up.

### Materials and Assessment

The 5-hour baseline-examination in the study center comprised evaluation of prevalent classical cardiovascular risk factors and clinical variables, a computer-assisted personal interview, laboratory examinations from a venous blood sample, blood pressure and anthropometric measurements. In general, all examinations were performed according to standard operating procedures by certified medical technical assistants.

### Measures

A shortened version of the somatic symptom module of the Patient Health Questionnaire (PHQ-15)^18,23,25^ assessed somatic symptoms. Three items were excluded from the original PHQ-15 scale: “menstrual cramps or other problems with your periods” applying only to female participants. As we aimed to assess the overlap with the Patient Health Questionnaire (PHQ-9)^16,26^, we further excluded the two items overlapping, “trouble sleeping” and “feeling tired or having low energy”. Severity of symptoms in the last four weeks were rated by three ordinal values (0 = not bothered at all; 1 = bothered a little; 2 = bothered a lot) by the study participants. Following the scoring of the PHQ-15, we added scores up to 0–24. In order to differentiate between different degrees of somatic symptom load, we divided symptom burden by quartiles (score from no 0–1, hardly 2–3, moderately 4–5, and >5 strongly). We tested the psychometric property of the PHQ-12 via confirmatory factor analysis. The model fit indices of the 1-factor model turned out to be acceptable to good: c^2^(df) = 1153, p < 0.001, CFI = 0.92, RMSEA = 0.05 [CI 0.05–0.06], SRMR = 0.06.

Depression was assessed with the Patient Health Questionnaire (PHQ-9). Caseness at follow-up was defined by a score ≥ 10. Löwe *et al*.^26^ found a sensitivity of 81% and a specificity of 82% for depressive disorder determined by this cut-off.

Generalized anxiety was assessed with the two item short form of the GAD-7 (Generalized Anxiety Disorder GAD – 2 Scale). A sum score of 3 and more (range 0–6) out of these two items indicates generalized anxiety with good sensitivity (86%) and specificity (83%)^27^.

Panic disorder was screened with the brief PHQ panic module. Caseness was defined if at least two of the first four PHQ panic questions are answered with ‘yes’^28^.

The German version of the Mini-Social Phobia Inventory (Mini-Spin)^29^ was used to detect social anxiety. Utilizing a cut-off score of 6 (range 0–12), the Mini-Spin is supposed to separate between individuals with generalized social anxiety disorder and controls with good sensitivity (89%) and specificity (90%).

Loneliness was assessed by a single item “I am frequently alone/have few contacts” rated as 0 = no, does not apply, 1 = yes it applies, but I do not suffer from it, 2 = yes, it applies, and I suffer slightly, 3 = yes, it applies, and I suffer moderately, 4 = yes, it applies, and I suffer strongly. Loneliness was recoded combining 0 and 1 = no loneliness or distress; 2 = slight, 3 = moderate, and 4 = severe loneliness^30^.

Participants rated their overall health in a single item from 1 = “very good” to 5 = “bad”.

*Physical activity* was inquired with the Short Questionnaire to Assess Health-Enhancing Physical Activity (SQUASH)^31,32^. The SQUASH captures commuting, leisure time, household, work and school activities. Sleeping, lying, sitting and standing were classified as inactivity. Active sports was presented in quartiles with Q1 denominating the lowest and Q4 the highest quartile of physical activity.

### Computer-assisted Personal Interview

During the computer-assisted personal interview participants were asked whether they had ever received the definite diagnosis of any depressive disorder or any anxiety disorder by a physician. The presence of coronary heart disease was assessed by the question: ‘Were you diagnosed with a stenosis of your coronary vessels?’ Self-reported myocardial infarction (MI), heart failure (HF), stroke, deep vein thrombosis (DVT), pulmonary embolism (PE), and peripheral arterial disease (PAD) were summarized as cardiovascular risk disease (CVD); cancer and COPD were assessed the same way.

Diabetes was defined in individuals with a definite diagnosis of diabetes by a physician or a blood glucose level of ≥126 mg/dl in the baseline examination after an overnight fast of at least 8 hours or a blood glucose level of >200 mg/dl after a fasting period of 8 hours.

Cardiovascular risk factors were defined as follows: Smoking was dichotomized into non-smokers (never smoker and ex-smoker) and current smokers (occasional and regular smokers). Obesity was defined as a body mass index > = 30 kg/m^2^. Diabetes was defined in individuals with a definite diagnosis of diabetes by a physician or a blood glucose level of > = 126 mg/dl in the baseline examination after an overnight fast of at least 8 hours or a blood glucose level of >200 mg/dl after a fasting period of 8 hours. Obesity was defined as a body mass index ≥30 kg/m² (0 = no, 1 = yes). Alcohol consumption was measured in gram per day; alcohol abuse was defined as daily consumption ≥60 mg for men and ≥40 mg for women.

Socioeconomic status was defined according to Lampert, Kroll, Müters, and Stolzenberg (2009)^33^ from 3 (lowest socioeconomic status) to 21 (highest socioeconomic status) based on education, profession and income.

### Statistical analysis

Descriptive analyses were performed as absolute and relative proportions for categorical data, means and standard deviations for continuous variables with approximately normal distribution and median with quartiles if not fulfilling this criterion.

In order to identify determinants of somatic symptom load, we used stepwise multiple regression analysis. In the regressions we used the raw score on the PHQ. Model 1 included sociodemographic, 2 additionally cardiovascular risk factors, 3 mental distress, and 4 somatic diseases. Analyses were done for all subjects and for men and for women separately. In the first model we included all sociodemographic, behavioral and psychological variables. In the second model we additionally included major somatic disorders (diabetes, CVD, COPD, cancer) in order to determine their additional contribution to somatic symptoms when the sociodemographic, behavioral and psychological variables are also in the equation.

Due to the large sample size, p-values should be interpreted with caution and in connection with effect estimates. Thus, all p-values should be regarded as a continuous parameter that reflect the level of statistical evidence and are therefore reported exactly. Statistical analysis was carried out using R version 3.3.1.

## Results

**Figure 1:**
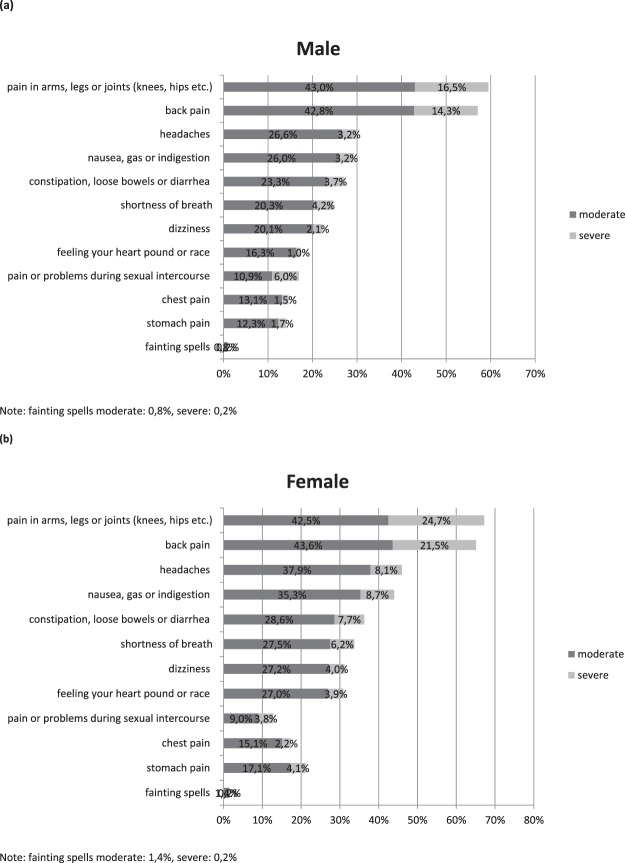
Prevalences of individual symptoms of the PHQ-12 for moderate and severe degree, separately for men (**a**) and for women (**a**,**b**) men (*N*_*range*_ = 3944–4064), (**b**) women (*N*_*range*_ = 3729–3881).

Figure 1 describes the prevalences of individual symptoms of the PHQ-12 in increasing order, for moderate and severe degree, separately for men (a) and for women (b).

As Fig. 1 shows, pain in arms, legs and joints was by far the most frequent symptom affecting 67% of the women and 60% of the men, followed by back pain affecting 65% of women and 58% of men. In descending order came headaches, nausea, constipation/diarrhea, shortness of breath, dizziness and heart racing or pounding. Women consistently reported more symptoms than men, except for pain or problems during sexual intercourse, where men exceed women slightly (17% vs. 13%).

The items excluded for overlap with depression (not in the figure) were the most frequent symptoms: feeling tired affecting ¾th of the women and 64% of the men (25.1% vs. 17.6% bothered a lot), followed by trouble sleeping affecting two thirds of men and women alike (14.9% vs. 10.2% bothered a lot).

### Determinants of somatic symptom load

Table 1 shows sample characteristics according to different degrees of symptom load based on the PHQ-12 (no, mild, moderate, severe).

**Table 1:** Characteristics of the sample GHS (*N* = 7974).

	PHQ12 0–1 (*N* = 2825) 35.4%	PHQ12 2–3 (*N* = 1978) 24.8%	PHQ12 4–5 (*N* = 1392) 17.5%	PHQ12 > 5 (*N* = 1779) 22.3%	p
*Sociodemographic*					
Sex (% women)	38.7 (1092)	47.0 (929)	54.3 (756)	63.0 (1121)	<**0.0001**
Age (years) (SD)	58.5 (10.6)	59.6 (10.5)	60.4 (10.6)	61.0 (10.5)	<**0.0001**
SES (SD)	13.72 (4.49)	13.17 (4.40)	12.56 (4.40)	11.72 (4.23)	<**0.0001**
Partnership (%)	87.5 (2330/2662)	86.2 (1600/1857)	86.3 (1116/1293)	83.4 (1388/1664)	**0.00037**
Unemployment (%)	0.9 (24/2823)	1.0 (20/1975)	1.5 (21/1389)	2.0 (36/1777)	**0.00028**
*Additional cardiovascular risk factors*					
Hypertension (%)	51.4 (1451/2824)	53.0 (1048/1976)	58.4 (812/1391)	59.1 (1052/1779)	<**0.0001**
Obesity (%)	20.7 (586/2825)	25.7 (508/1978)	27.0 (375/1390)	33.7 (600/1778)	<**0.0001**
Smoking (%)	14.5 (410/2825)	15.2 (300/1977)	15.0 (209/1392)	16.7 (297/1777)	0.063
FH of MI/Stroke (%)	20.5 (580/2825)	22.0 (436/1978)	24.0 (334/1392)	28.8 (513/1779)	<**0.0001**
Alcohol abuse (%)	2.2 (61/2825)	2.4 (48/1978)	1.9 (27/1392)	2.3 (41/1779)	0.96
Social support	16.00	15.00	15.00	14.00	<**0.0001**
Life events	0.40	0.40	0.40	0.60	<**0.0001**
*Mental distress*					
PHQ9 ≥ 10 (%)	1.2 (34/2821)	4.8 (94/1978)	8.6 (119/1391)	23.2 (412/1777)	<**0.0001**
GAD2 ≥ 3 (%)	1.5 (43/2818)	3.9 (77/1977)	6.6 (92/1390)	17.4 (309/1774)	<**0.0001**
Panic (%)	1.6 (45/2815)	3.4 (67/1972)	4.9 (68/1380)	13.2 (233/1760)	<**0.0001**
Social Phobia (%)	2.0 (56/2810)	3.7 (74/1974)	4.8 (67/1387)	11.6 (206/1770)	<**0.0001**
Loneliness (%)	5.8 (163/2799)	8.9 (175/1964)	11.2 (154/1371)	20.1 (355/1762)	<**0.0001**
*Somatic diseases*					
Diabetes (%)	9.2 (260/2812)	9.2 (182/1973)	10.6 (146/1383)	14.2 (252/1772)	<**0.0001**
CVD (%)	9.2 (260/2817)	13.6 (268/1974)	14.9 (206/1380)	23.4 (414/1771)	<**0.0001**
COPD (%)	3.4 (95/2823)	6.4 (126/1976)	7.2 (100/1389)	14.6 (260/1778)	<**0.0001**
Cancer (%)	10.1 (284/2822)	13.5 (267/1977)	12.4 (173/1390)	15.6 (278/1778)	<**0.0001**

Note: Alcohol abuse: >60/40. Life events: last 12 months. Social Phobia: Mini-Spin ≥6.

As Table 1 shows, 35% of participants were in the first (no) and 25% in the second (mild), 18% in the third (moderate) and 22% in the fourth quartile (severe somatic symptoms). Regarding sociodemographic variables, increasing severity was associated with a greater proportion of women, higher age, and unemployment, whereas SES, income and the presence of a partnership decreased. There was an increase of hypertension, obesity, and family history of myocardial infarction (MI). Strong relationships were found for depression: one percent of participants without high somatic symptoms were depressed vs. one fourth of those with high symptom load. Similar patterns were found for generalized anxiety disorder, panic and social phobia. Whereas only 5% of participants with low somatic symptom load were lonely, this applied to one in five with a high symptom load. There was a decrease of social support and an increase of life events with increasing somatic symptoms. Increases were also found for diabetes, cardiovascular diseases (including MI, stroke, heart failure), COPD and cancer.

Table 2 shows multivariate linear regression analysis on the PHQ-12 score. We performed stepwise hierarchical regression. Entering sociodemographic factors (model 1), female sex, lower SES, and unemployment accounted for 6.3% of the variance. When cardiovascular risk factors were added, obesity, smoking and family history of myocardial infarction/stroke were significant predictors (explaining an addition 1.4% of variance). Variance explained increased to 25%, when distress (life events, current depression, generalized anxiety, panic, social phobia and loneliness) and social support as a protective factor were entered as significant predictors into the model, and smoking was no longer significant.

**Table 2:** Multiple linear regression models (overall).

	Model 1 sociodemographic (*N* = 7244)		Model 2 additional cardiovascular risk factors (*N* = 7238)		Model 3 mental distress (*N* = 7076)		Model 4 somatic diseases (*N* = 7022)	
	b	P	b	p	b	p	b	p
R^2^, F-value (df1, df2)	0.0633, 97.8(5,7238)	**<0.0001**	0.0771, 60.4(10,7227)		0.25, 138(17,7058)	**<0.0001**	0.279, 129(21,7000)	**<0.0001**
Sex (Women)	1.16	**<0.0001**	1.19	**<0.0001**	0.976	**<0.0001**	1.04	**<0.0001**
Age (10 y)	0.169	**<0.0001**	0.155	**0.00015**	0.386	**<0.0001**	0.235	**<0.0001**
SES	−0.102	**<0.0001**	−0.0855	**<0.0001**	−0.0804	**<0.0001**	−0.0740	**<0.0001**
Partnership	−0.0557	0.62	−0.0707	0.53	0.546	**<0.0001**	0.514	**<0.0001**
Unemployment	1.70	**<0.0001**	1.58	**<0.0001**	0.655	**0.044**	0.627	0.052
Hypertension			0.143	0.095	0.172	**0.027**	0.135	0.078
Obesity			0.698	**<0.0001**	0.551	**<0.0001**	0.424	**<0.0001**
Smoking			0.316	**0.0040**	0.152	0.13	0.120	0.22
FH of MI/Stroke			0.472	**<0.0001**	0.358	**<0.0001**	0.279	**0.00059**
Alcohol abuse			−0.0662	0.80	−0.245	0.29	−0.165	0.47
Social support					−0.0942	**<0.0001**	−0.0921	**<0.0001**
Life events last 12 months					0.688	**<0.0001**	0.652	**<0.0001**
PHQ9 ≥ 10					2.63	**<0.0001**	2.57	**<0.0001**
GAD2 ≥ 3					1.30	**<0.0001**	1.25	**<0.0001**
Panic					1.71	**<0.0001**	1.62	**<0.0001**
Social Phobia					0.706	**<0.0001**	0.674	**<0.0001**
Loneliness					0.570	**<0.0001**	0.562	**<0.0001**
Diabetes							0.170	0.15
CVD							1.13	**<0.0001**
COPD							1.44	**<0.0001**
Cancer							0.374	0.00046

Note: b: unstandardized regression coefficient. p: p-value. Alcohol abuse: >60/40. Life events last 12 months: per 5 events. Social Phobia: Mini-Spin ≥6.

When somatic illnesses were added, unemployment and hypertension were no longer statistically significant. The presence of cardiovascular disease, COPD and cancer were significantly predictive of somatic symptom load. The proportion of variance explained, however, only increased by another 2.9% to a total of 27.9%.

Table 3 shows multiple logistic regression models (overall) with sex interaction. As shown by the interaction between sex and depression, depression plays a smaller role for somatic symptom reporting in women vs. men (smaller OR in tab. 3 and smaller b in the separate analyses in Supplementary Table S1 that can be found online).

**Table 3:** Multiple logistic regression models (Overall): with sex interaction.

	N	OR	L 95%CI	U 95%CI	p-value
PHQ12 > = 10	**7022(586 events)**				
Sex		5.034	2.384	11.000	<**0.0001**
Age [10 y]		1.292	1.048	1.597	**0.017**
SES		0.929	0.891	0.967	**0.00044**
Partnership		1.973	1.149	3.524	**0.017**
Unemployment		3.829	1.537	8.781	**0.0024**
I: Age [10 y]*Sex(Women)		0.844	0.654	1.089	0.19
I: SES*Sex(Women)		1.003	0.951	1.057	0.92
I: Partnership*Sex(Women)		0.772	0.399	1.456	0.43
I: Unemployment*Sex(Women)		0.294	0.077	1.106	0.070
Hypertension		1.354	0.927	2.001	0.12
Obesity		1.363	0.947	1.951	0.093
Smoking		1.276	0.810	1.968	0.28
FH of MI/Stroke		1.469	1.019	2.095	**0.036**
Alcohol abuse (>60/40)		0.711	0.232	1.756	0.50
I: Hypertension*Sex(Women)		0.828	0.517	1.317	0.43
I: Obesity*Sex(Women)		0.814	0.519	1.279	0.37
I: Smoking*Sex(Women)		0.718	0.412	1.260	0.24
I: FH of MI/Stroke*Sex(Women)		0.908	0.586	1.414	0.67
I: Alcohol abuse (>60/40)*Sex(Women)		1.361	0.351	5.563	0.66
PHQ9 > = 10		7.164	4.584	11.151	<**0.0001**
GAD2 > = 3		1.716	1.002	2.893	**0.046**
Panic		2.826	1.642	4.778	**0.00013**
Social Phobia (Mini-Spin > = 6)		1.417	0.793	2.481	0.23
Loneliness		1.661	1.005	2.702	**0.044**
Social support		0.930	0.888	0.974	**0.0021**
Life events last 12 months [per 5 events]		1.281	0.918	1.770	0.14
I: PHQ9 > = 10*Sex(Women)		0.436	0.248	0.766	**0.0039**
I: GAD2 > = 3*Sex(Women)		1.281	0.671	2.464	0.46
I: Panic*Sex(Women)		0.858	0.452	1.644	0.64
I: Social Phobia (Mini-Spin > = 6)*Sex(Women)		0.923	0.460	1.868	0.82
I: Loneliness*Sex(Women)		0.888	0.491	1.621	0.70
I: Social support*Sex(Women)		1.032	0.975	1.093	0.27
I: Life events last 12 months [per 5 events]*Sex(Women)		0.973	0.649	1.465	0.89
Diabetes		1.195	0.771	1.821	0.42
CVD		1.990	1.362	2.894	**0.00034**
COPD		2.117	1.272	3.410	**0.0028**
Cancer		1.088	0.672	1.715	0.72
I: Diabetes*Sex(Women)		0.944	0.525	1.699	0.85
I: CVD*Sex(Women)		0.946	0.573	1.560	0.83
I: COPD*Sex(Women)		1.182	0.661	2.157	0.58
I: Cancer*Sex(Women)		1.239	0.705	2.210	0.46

Supplementary Table S1 can be found online and shows separate analyses for men (a) and women (b). Age was positively associated with somatic symptom load. SES and unemployment were associated with somatic symptom load in both, men and women (model 1). In model 2, smoking and hypertension were predictors in men, but not in women. In model 3, unemployment and obesity were no longer significant in women. In model 4, cancer was only significant in women (504 women vs. 490 men suffered from cancer).

## Discussion

The purposes of the paper were to assess the somatic symptom load in a community sample of men and women from middle to old age (40–80 years) and to identify psychosocial and somatic determinants of symptom reporting. Based on the PHQ-15, one of the mostly used assessments, however, we excluded 3 items in order to avoid confounding. Two items overlapping with the depression measure used (PHQ-9) were excluded along with the menstruation complaints item which biases sex comparisons. Based on the PHQ-12, we found a high rate of somatic complaints, and the order of symptoms was comparable to Hinz *et al*.^1^. Our study adds to the existing research since we identify the somatic symptom burden without confounding of symptoms of depression and sex. Interestingly, the two items excluded for overlap with depression were the most frequent symptoms: feeling tired affecting ¾th of the women and 64% of the men, followed by trouble sleeping affecting two thirds of men and women alike (14.9% vs. 10.2% bothered a lot). Including menstrual complaints in a total sum score has created a sex bias, which has remained surprisingly unreflected in previous reports on the PHQ-15. While our findings support the use of the PHQ for assessing somatic symptoms in the general population, further research should consider the strong overlap of somatic symptoms with depression and sex in the differential diagnosis of the new diagnostic entity of “somatic symptom disorder”. When confounding has been reduced, psychological factors have remained the strongest predictors of somatic symptoms.

Corresponding to Hinz *et al*.^1^ who used the PHQ-15 for their assessment, about two thirds of the women and 60% of the men reported pain in arm, legs and joints, respectively back pain. In declining order came headaches, nausea, constipation/diarrhea, shortness of breath, dizziness and heart racing or pounding. Women consistently reported more symptoms than men, except for pain or problems during sexual intercourse, where men exceed women slightly.

When stratified by severity, symptoms increased with sex and age, lower SES, the lack of a partnership and unemployment. Cardiovascular risk factors of smoking, hypertension, and obesity were also associated with symptom reporting. The greatest differences were found for depression, generalized anxiety, panic, social phobia and loneliness. Social support declined with symptom reporting, while life events increased. Symptom-reporting was associated with the presence of somatic disorders, particularly for COPD, but also for CVD, cancer and diabetes.

In a stepwise hierarchical regression model, demographic factors (female sex, low SES, the lack of a partnership, unemployment) explained 6% of variance. Additional cardiovascular risk factors (obesity, family history of MI) added 1.4%, including psychosocial factors added 17% (lack of social support, life events, loneliness and mental distress) and somatic diseases (CVD, COPD, cancer) another 3% of variance explained. The total model explained 28% of variance.

Our findings highlight the multifactorial nature of somatic symptom reporting. Psychosocial factors include demographic characteristics such as female sex and low SES, and the absence of a partnership. In addition to mental distress, as in Le Roy *et al*.^17^, loneliness was an important factor. While they explained less overall variance, somatic disease and related risk factors such as obesity should not be overlooked. Unemployment and hypertension were only significant predictors in men, whereas loneliness and cancer predicted symptom load in women.

As shown by the interaction between sex and depression, depression plays a smaller role for somatic symptom reporting in women vs. men. This raises interesting questions on the significance of gendered expression of depression for somatic symptom reporting^34^.

Future analyses differentiating psychosocial and somatic contributions to somatic symptom load in men and women should use the PHQ-12 item version in order to avoid confounding. Different relevance of predictors of somatic symptom load in men and women needs to be considered^3,28,30,31,33,34^.

### Benefits and limitations

The study used a large sample with a comprehensive set of assessmen ts across a large age range with equal strata of men and women in each age group. In order to reduce confounding, we eliminated two items and sex bias by deleting menstrual complaints. Differentiating degrees of somatization based on quartiles may be seen as arbitrary, and neither the cut-offs nor the total score of the PHQ-12 are comparable to other studies using the PHQ-15. We could not assess health-related preoccupation and anxiety, which was introduced into the DSM-V as part of the diagnosis of somatic symptom disorder after the start of the survey. We did not analyse utilization of health care as an important criterion of high somatic symptom load. As we analyzed cross-sectional data, we cannot determine causal effects.

## Supplementary information

Supplementary Table S1

## Data Availability

For approved reasons, some access restrictions apply to the data underlying these findings. Data sets contain identifying participant information, which is not suitable for public deposition. Access to the local database is available upon request to the corresponding author.
