# Supplementary material for: Somatic symptom load in men and women from middle to high age in the Gutenberg Health Study - association with psychosocial and somatic factors
Source: Sci Rep. 2019 Mar 14;9:4610. doi: 10.1038/s41598-019-40709-0 (PMC6418216; doi:10.1038/s41598-019-40709-0)
Supplement: Supplementary file 1 — Supplementary Table S1 [file 41598_2019_40709_MOESM1_ESM.pdf]

Somatic symptom load in men and women from middle to high age in the Gutenberg Health Study - association with psychosocial and somatic factors

Manfred E. Beutel, MD, Jörg Wiltink, MD, Jasmin Ghaemi Kerahrodi, MD, Ana N. Tibubos, PhD, Elmar Brähler, PhD, Andreas Schulz, PhD, Philipp Wild, MD, Thomas Münzel, MD, Karl Lackner, MD, Jochem König, PhD, Norbert Pfeiffer, MD, Matthias Michal, MD, Michaela Henning, MD

Supplementary Table 1a: Multiple linear regression models (men)

	**Model 1 sociodemographic** (*N* = 3811)		**Model 2 additional cardiovascular risk factors** (*N* = 3808)		**Model 3 mental distress** (*N* = 3741)		**Model 4 somatic diseases** (*N* = 3713)	
	b	P	b	p	b	p	b	p
R^2, F-value (df1, df2)	.0373, 36.9 (4, 3806)	**<.0001**	.0532, 23.7 (9, 3798)		.231, 69.9 (16, 3724)	**<.0001**	.258, 64.3 (20, 3692)	**<.0001**
Age (10y)	0.231	**<.0001**	0.221	**<.0001**	0.417	**<.0001**	0.259	**<.0001**
SES	-0.0967	**<.0001**	-0.0827	**<.0001**	-0.0754	**<.0001**	-0.0707	**<.0001**
Partnership	-0.0368	.82	-0.0852	.59	0.550	**.00031**	0.532	**.00040**
Unemployment	2.01	**<.0001**	1.84	**<.0001**	1.30	**.0011**	1.34	.00068
Hypertension			0.258	**.017**	0.234	**.018**	0.193	**.049**
Obesity			0.618	**<.0001**	0.550	**<.0001**	0.451	**<.0001**
Smoking			0.389	**.0052**	0.251	**.047**	0.189	.13
FH of MI/Stroke			0.438	**.00022**	0.321	**.0029**	0.259	**.015**
Alcohol abuse			-0.320	.26	-0.245	.35	-0.165	.52
Social support					-0.103	**<.0001**	-0.101	**<.0001**
Life events last 12 months					0.666	**<.0001**	0.662	**<.0001**
PHQ9 ≥ 10					3.16	**<.0001**	3.13	**<.0001**
GAD2 ≥ 3					1.00	**<.0001**	0.950	**<.0001**
Panic					1.59	**<.0001**	1.50	**<.0001**
Social Phobia					0.832	**.0011**	0.751	**.0029**
Loneliness					0.287	.13	0.274	.14
Diabetes							0.141	.31
CVD							1.01	**<.0001**
COPD							1.33	**<.0001**
Cancer							0.202	.15

Note: b: unstandardized regression coefficient. p: p-value. Alcohol abuse: >60/40. Life events last 12 months: per 5 events. Social Phobia: Mini-Spin≥6.

Supplementary Table 1b: Multiple linear regression models (women)

	**Model 1 sociodemographic** (*N* = 3433)		**Model 2 additional cardiovascular risk factors** (*N* = 3430)		**Model 3 mental distress** (*N* = 3335)		**Model 4 somatic diseases** (*N* = 3309)	
	b	p	b	p	b	p	b	p
R^2, F-value (df1, df2)	.0225, 19.8 (4, 3428)	**<.0001**	.0369, 14.5 (9, 3420)		.222, 59.1 (16, 3318)	**<.0001**	.254, 56 (20, 3288)	**<.0001**
Age (10y)	0.0897	.14	0.0753	.26	0.346	**<.0001**	0.222	**.00060**
SES	-0.109	**<.0001**	-0.0916	**< .0001**	-0.0860	**<.0001**	-0.0767	**<.0001**
Partnership	-0.0900	.58	-0.0780	.63	0.532	**.00043**	0.505	**.00068**
Unemployment	1.26	**.032**	1.18	**.045**	-0.252	.64	-0.395	.47
Hypertension			0.0243	.86	0.101	.41	0.0520	.67
Obesity			0.797	**< .0001**	0.564	**<.0001**	0.402	**.0024**
Smoking			0.229	.19	0.0311	.84	0.0418	.79
FH of MI/Stroke			0.511	**.00022**	0.409	**.0011**	0.296	**.017**
Alcohol abuse			0.434	.38	-0.290	.52	-0.169	.70
Social support					-0.0854	**.0011**	-0.0848	**<.0001**
Life events last 12 months					0.741	**.0011**	0.670	**<.0001**
PHQ9 ≥ 10					2.18	**.0011**	2.11	**<.0001**
GAD2 ≥ 3					1.58	**.0011**	1.52	**<.0001**
Panic					1.76	**.0011**	1.67	**<.0001**
Social Phobia					0.655	**.0061**	0.668	**.0045**
Loneliness					0.757	**.0011**	0.759	**<.0001**
Diabetes							0.205	.33
CVD							1.32	**<.0001**
COPD							1.54	**<.0001**
Cancer							0.554	**.00067**

Note: b: unstandardized regression coefficient. p: p-value. Alcohol abuse: > 60/40. Life events last 12 months: per 5 events. Social Phobia: Mini-Spin≥6.
